# Supplementary material for: Ascertaining cells’ synaptic connections and RNA expression simultaneously with barcoded rabies virus libraries
Source: Nat Commun. 2022 Nov 16;13:6993. doi: 10.1038/s41467-022-34334-1 (PMC9668842; doi:10.1038/s41467-022-34334-1)
Supplement: Supplementary file 1 — Supplementary Information [file 41467_2022_34334_MOESM1_ESM.pdf]

Supplementary Information

Ascertaining cells' synaptic connections and RNA expression simultaneously with barcoded rabies virus libraries

Arpiar Saunders[1,2,4*], Kee Wui Huang[3], Cassandra Vondrak[1,2], Christina Hughes[1,2], Karina Smolyar[1,2], Harsha Sen[1,2], Adrienne C. Philson[3], James Nemesh[1,2], Alec Wysoker[1,2], Seva Kashin[1,2], Bernardo L. Sabatini[3] and Steven A. McCarroll[1,2*]

Contents:

Supplementary Figures 1-10

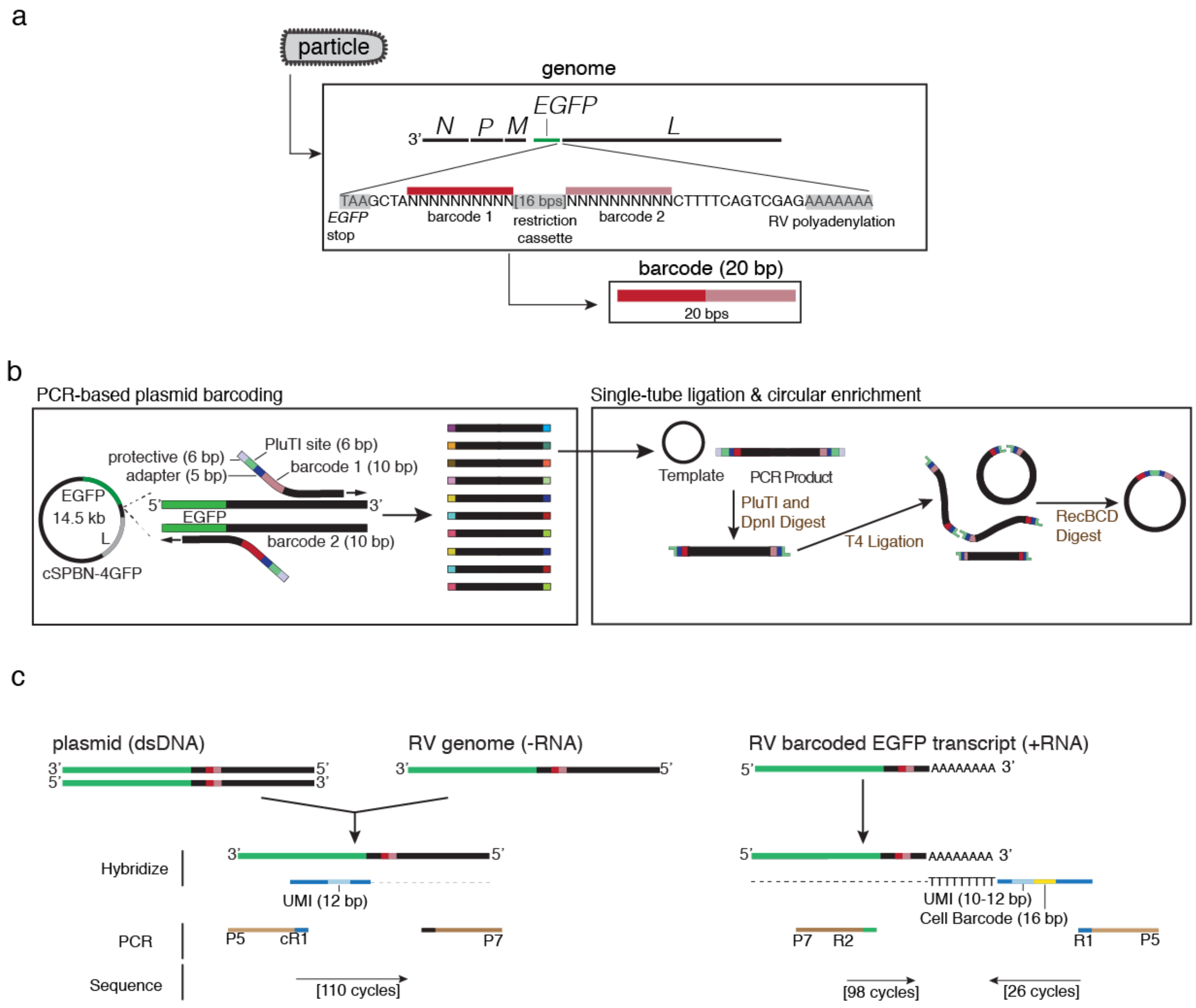

Supplementary Figure 1. Molecular workflows for PCR-based plasmid barcoding and UMI-based VBC quantification across plasmids, anti-sense rabies virus genomes and mRNAs. a. Schematic showing the VBC cassette integrated into the rabies genome. The 20 bp VBC consists of two 10 bp barcodes linked by a restriction cassette and sits 13 bps away from the rabies polyadenylation signal. **b.** Diagram describing the novel PCR-based strategy for barcoding DNA plasmids as applied to the cDNA version of the SAD-B19 RV*dG*-EGFP genome (pSPBN-4GFP). Forward and reverse primers containing 10 bp randomers - tailed by 17 bps of containing adaptor sequence, the PluTI site and protective bases – are targeted to adjacent regions of the template plasmid. Amplification results in a collection of linear dsDNA molecules each of which contain a unique combination of terminal 10 bp barcodes, which are then circularized and enriched (versus both template plasmid and remaining linear products) through a single-pot reaction. **c.** Illumina sequencing-based strategy for quantifying plasmid and anti-sense genomic barcodes (*left*) or barcoded *EGFP* mRNA (*right*) using unique molecular identifiers (UMIs). UMI-containing oligonucleotides (UMI = 12 bp randomer) with a shared PCR handle are hybridized adjacent to and then polymerized through the barcode cassette on ssDNA or anti-sense RNA genomes. The UMI-tagged ssDNA molecules are then selectively PCR amplified using primers that contain Illumina P5 and P7 sites and sequenced on an Illumina flow cell such that 110 Read 1 cycles cover the barcode cassette. Barcoded *EGFP* mRNA is selectively amplified from sc-cDNA using P5 and P7 containing primers and sequenced on an Illumina flowcell such that 26-28 Read 1 cycles that capture the 16 bp Cell Barcode and 10-12 bp UMI introduced by 10x Chromium v2 or v3 chemistry and 98 Read 2 cycles which extend through the barcode cassette (Methods). Source data are provided as a Source Data file.

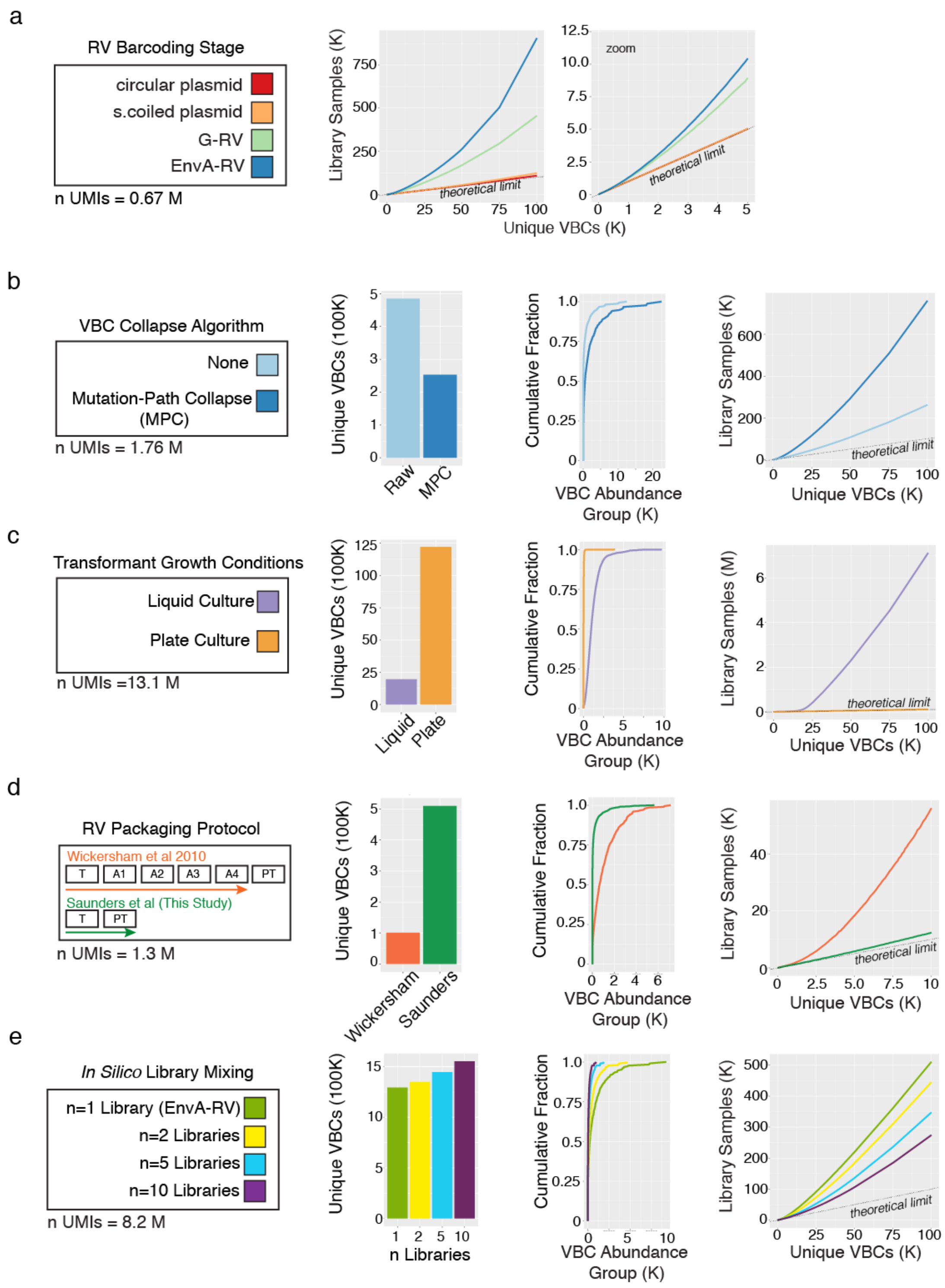

Supplementary Figure 2. Accurate and systematic quantification of VBC abundances guides optimized protocols for plasmid barcoding and barcoded rabies virus packaging. a. Longitudinal assessment of VBC diversity across each stage of rabies virus packaging protocol, as assayed through the

library sampling procedure in which total library samples are plotted against the number of unique VBCs ascertained from each sample. (Companion data to Fig. 1f,g. Plot includes data from Fig. 1f along with additional conditions). The dotted line shows maximum theoretical diversity (in which every drawn VBC is unique). **b-e**. Quantification of VBC abundance and diversity across various protocol conditions (sampled with equivalent UMIs, *far left*) by plotting (*from left to right*) total unique VBCs; cumulative distribution of UMIs by abundance group (as in Fig. 1f); and number of unique VBCs ascertained from a given number of library samples (as in **a** above). **b.** The effect of "mutation-path collapse" (MPC), an informatic approach implemented to help account for artifactual inflation of barcodes driven by mutations to barcode sequences incurred during library amplification or sequencing (Methods). **c.** The effect of *E.coli* growth conditions (plated or liquid culture) after transformation with circular barcoded plasmid library. Barcodes were sampled from super-coiled plasmid DNA. **d.** The effect of rabies virus packaging protocols, comparing the widely used Wickersham et al. 2010 protocol versus the barcode diversity optimized protocol reported in this study (Saunders et al. 2021). Barcodes were sampled from anti-sense genomes extracted from EnvA-pseudotyped libraries. **e**. The effect of combining different numbers of independent and equivalently diverse barcoded EnvA-pseudotyped libraries *in silico* (Methods). Source data are provided as a Source Data file.

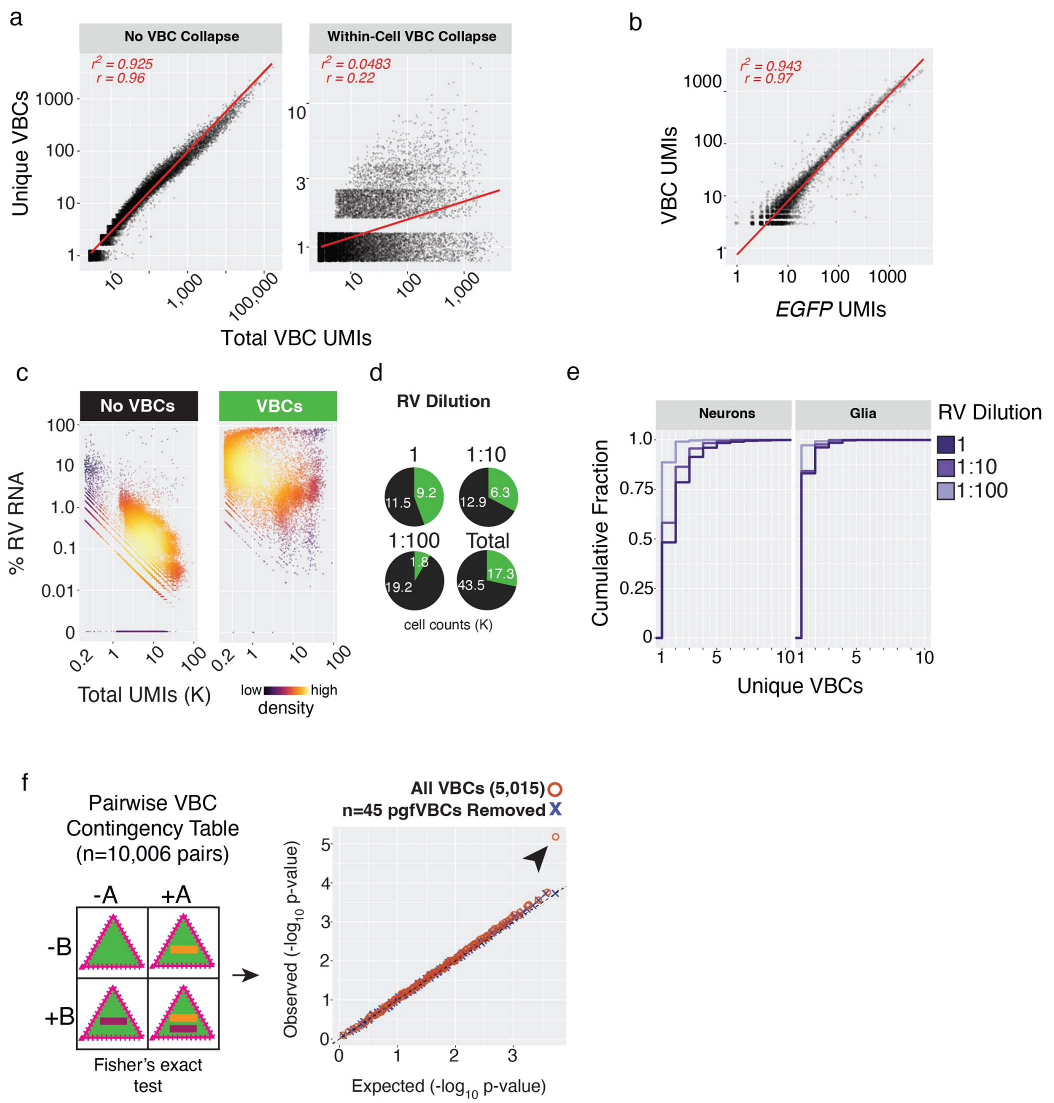

Supplementary Figure 3. Integrating host cell RNA and viral VBC data for thousands of individual starter cells relate how properties of barcoded rabies libraries behave across founder infections resolved by cell type. a. "Within-Cell VBC Collapse" corrects amplification and sequencing mutations in VBC sequences. *Left*, without correction (r = 0.96). *Right*, following "Within-Cell VBC Collapse" (r = 0.22). **b.** UMI counts for VBCs (3' *EGFP* UTR sequencing) and the *EGFP* mRNAs (host cell RNA sequencing) are highly correlated (r = 0.97; n = 6,979 cells, a single experiment). **c-e.** scRNA profiles ascertained from cultured brain cells expressing *TVA* (but not rabies *G)* transduced with EnvA-RV*dG-EGFP*$_{VBC}$ at different concentrations ("1", MOI ~ 15; diluted "1:10", MOI ~ 1.5; "1:100", MOI ~ 0.15). **c.** VBCs are ascertained from infected ("VBCs"; n = 17,283) but not uninfected ("No VBCs"; n= 43,533) scRNA profiles across UMI counts and total viral RNA percentages. **d.** Cell percentages with ascertained VBCs across MOIs. **e.** Cumulative distribution of unique VBCs per cell (Neurons: 1 vs 1:10, p = 1.846e-12; 1 vs 1:100, p < 2.2e-16; 1:10 vs 1:100, p < 2.2e-16; Glia: 1 vs 1:10, p = 1; 1 vs 1:100, p = 0.02; 1:10 vs 1:100, p = 0.04. Two-sided Kolmogorov–Smirnov Test). **f.** Testing VBC independence. *Left*, contingency table cartoon comparing how two VBCs ("A", purple; "B", orange) occur together (+A/+B), independently (+A or +B), or are unobserved (-A/-B). VBCs paired more than chance (n=45 of 10,009 total pairs with Bonferroni-corrected p < 0.05, Fisher's Exact test) were flagged as putative genome fusions (pgf). *Right*, Q-Q plot comparing observed vs expected (based on randomized VBC pairs) p-values when all VBCs pairs were considered (orange) and after n=45 putative fusions were removed (purple). Major difference indicated by arrowhead. Boxes define the interquartile range and whiskers delineate 1.5 times this range. All data are from n=5 culture wells derived from n=3 independent primary cell cultures. Source data are provided as a Source Data file.

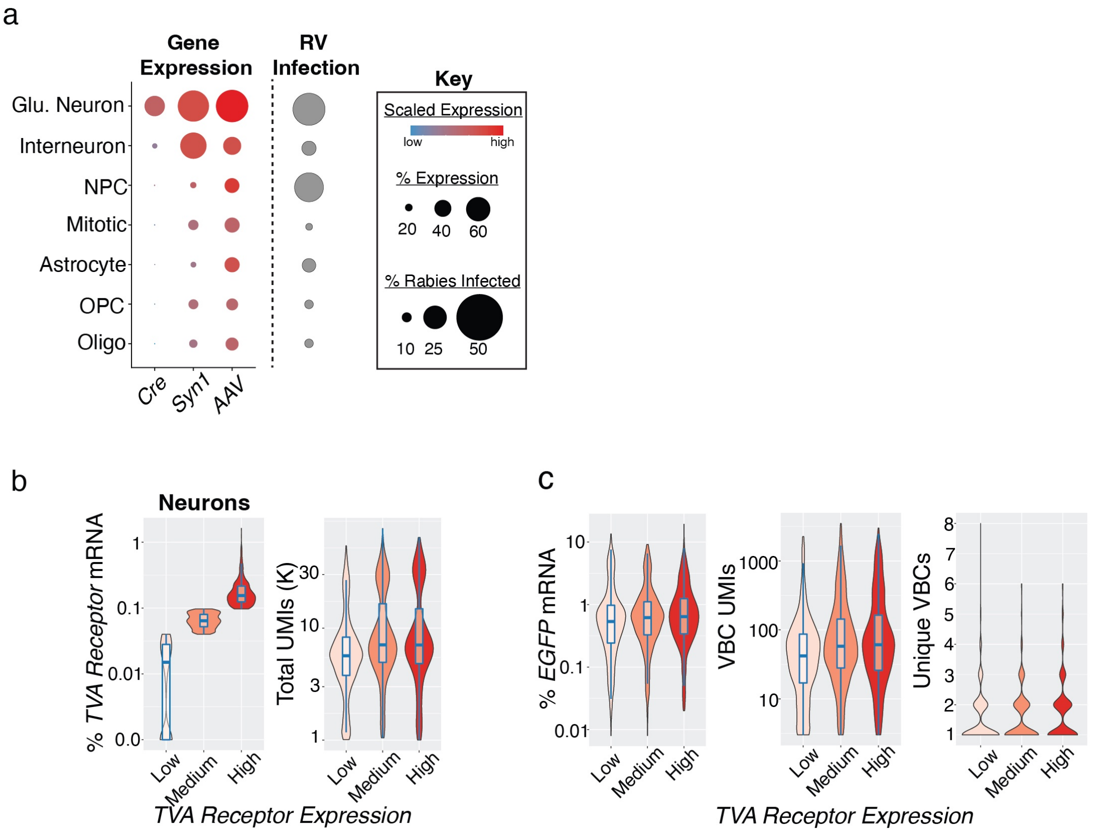

Supplementary Figure 4. TVA receptor expression dictates starter cell types yet differences in TVA receptor RNA exert little influence on downstream properties of RV infection. a. Dot plot analysis of RNAs that drive TVA receptor expression (*left*) compared to cell-type proportions of EnvA-RV*dG-EGFP*$_{\mathrm{VBC}}$ infected starter cells (*right*). Functional TVA receptor is expressed in cells in which CRE (driven by *Synapsin1* promoter AAV) recombines rAAV genomes expressing TCB (driven by the CAG promoter AAV). **b,c.** Differences in AAV mRNA – a proxy for TVA receptor expression – do not strongly influence RV infection properties. **b.** AAV RNA levels after stratification into neuron groups with low, medium and high expression (*left*). library sizes (*right*) were different across TVA receptor expression groups ($p < 2e-16$, one-way ANOVA). **c.** RV infection properties are similar across expression groups. The percentage of *EGFP* RNA driven by rabies virus expression (*left*) and the number of VBC UMIs (*middle*) were strongly associated with differences in library size but not AAV RNA expression levels (% EGFP: p = 0.65 & Partial R^2 = 0.000084, AAV RNA; $p < 2e-16$ & Partial R^2 = 0.03, Library Size. VBC UMIs: p = 0.02 & Partial R^2 = 0.002, AAV RNA; $p < 2e-16$ & Partial R^2 = 0.45, Library Size. Linear regression analysis). *Right*, the number of unique VBCs across expression groups were similar (Mean Unique VBCs: Low, 1.5; Medium, 1.6; High, 1.7). Unique VBC distributions were significantly difference (p = 1.379e-05, A Chi-square test) but the effect was weak (Cramer's V = 0.07). Source data are provided as a Source Data file.

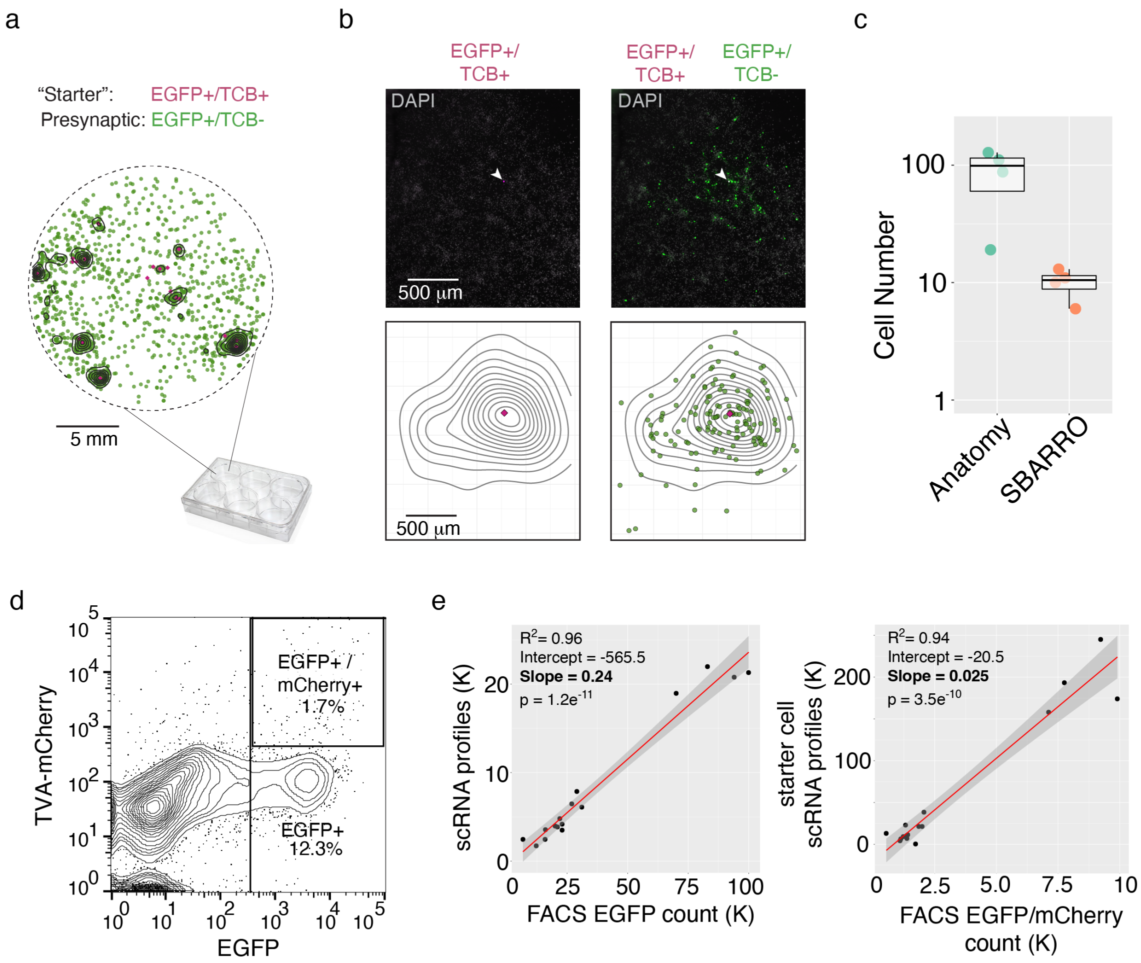

Supplementary Figure 5. Anatomy of monosynaptic rabies virus spread *in vitro*. a-b. Monosynaptic cell-to-cell spread events of rabies virus in cell cultures derived from dissociated embryonic mouse cortex exhibit stereotyped spatial patterning. In each culture well, a small subset of potential starter cells was endowed using a rAAV Cre-recombinase based strategy followed by transduction of EnvA-RV*dG-EGFP*$_{\text{VBC}}$. Fluorescent scans of whole culture wells distinguish the locations of these spatially sparse starter (EGFP+/TVA-mCherry+, magenta) and presynaptic cells (EGFP+/TVA-mCherry-, green)(Methods). **a.** Locations of starter and presynaptic cells from a representative culture well. Presynaptic cells tend to spatially cluster around starter cells, but are also observed at greater distances. Contours illustrate density of rabies virus infected cells. **b.** Higher magnification view of a single starter cell (*left*) and presynaptic cells in close proximity (*right*). *Top*, fluorescent images. *Bottom*, plot of extracted cell locations. **c.** A comparison of inferred presynaptic network sizes based on anatomical imaging (the number of clustered presynaptic cells) or SBARRO sequencing (the number of presynaptic scRNA profiles based on uCIPs). Anatomy data was from n=23 networks from n=3 cultures wells derived from n=3 primary cell culture experiments. SBARRO data was from n=3 culture wells from n=1 primary cell culture experiment. The largest n=4 networks from each modality were compared. Experiments were performed in parallel from neighboring culture wells grown from the same cell suspension. **d**. Example fluorescent signals used for FACS enrichment of EGFP+ cells and posthoc counting of EGFP+ versus EGFP+/TVA-mCherry+ cells upstream of scRNA-seq library generation. Contours represent particle density. Gated particle percentages are listed. **e.** Paired comparisons from n=16 culture wells of FACS-based cell counts and single-cell RNA profiles for all rabies virus infected EGFP+ cells (*left*) and for EGFP+/ TVA-mCherry starter cells (*right*). For both comparisons, linear regressions demonstrate that the number of FACS-counted cells was strongly predictive of SBARRO sampling (EGFP+ cells, R^2 = 0.96 and p = 1.2e-11; EGFP+/ TVA-mCherry, R^2 = 0.94 and p = 3.5e-10), with slope estimates suggesting ~24% of all FACS-counted EGFP+ cells were sampled and ~2.5% of EGFP+/ TVA-mCherry starter cells were identified (Methods). Shaded regions correspond to 95% confidence intervals. Source data are provided as a Source Data file.

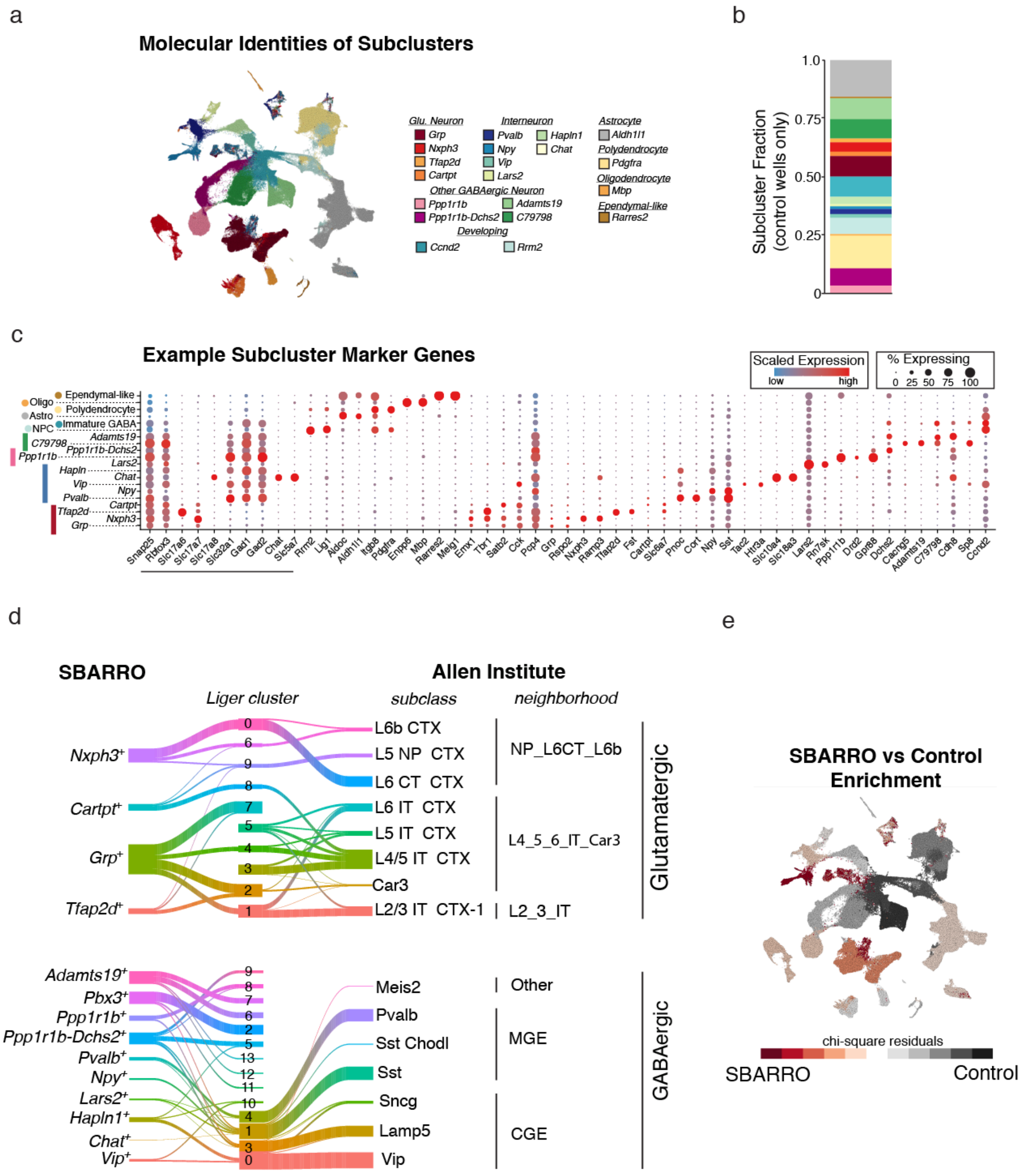

Supplementary Figure 6. Assigning molecular identities to SBARRO scRNA profiles. a. UMAP embedding color-coded by granular molecular subtypes (subclusters). b. Proportions of RNA profiles in each subcluster observed in RV uninfected control wells. c. Dotplot of example marker gene expression patterns across subcluster populations. Common markers for neurons and neuron types are underlined; additional genes pairs were selected for each population based on differential expression analysis (Methods). **d.** Sankey plots showing molecular homologies between scRNA profiles from SBARRO control cells *in vitro* and adult mouse cortex *in vivo*[18] following LIGER analysis of glutamatergic (SBARRO, n = 24,155 profiles; Allen Institute, n= 38,899) and GABAergic (SBARRO, n = 54,713 profiles; Allen Institute, n= 18,163) neurons (Methods). **e.** Quantifying enrichment or depletion of SBARRO libraries as compared to control scRNA profiles. Color-code shows chi-square residuals after the number of SBARRO/control RNA profiles within each coarse molecular population are compared to dataset totals (Methods). Source data are provided as a Source Data file.

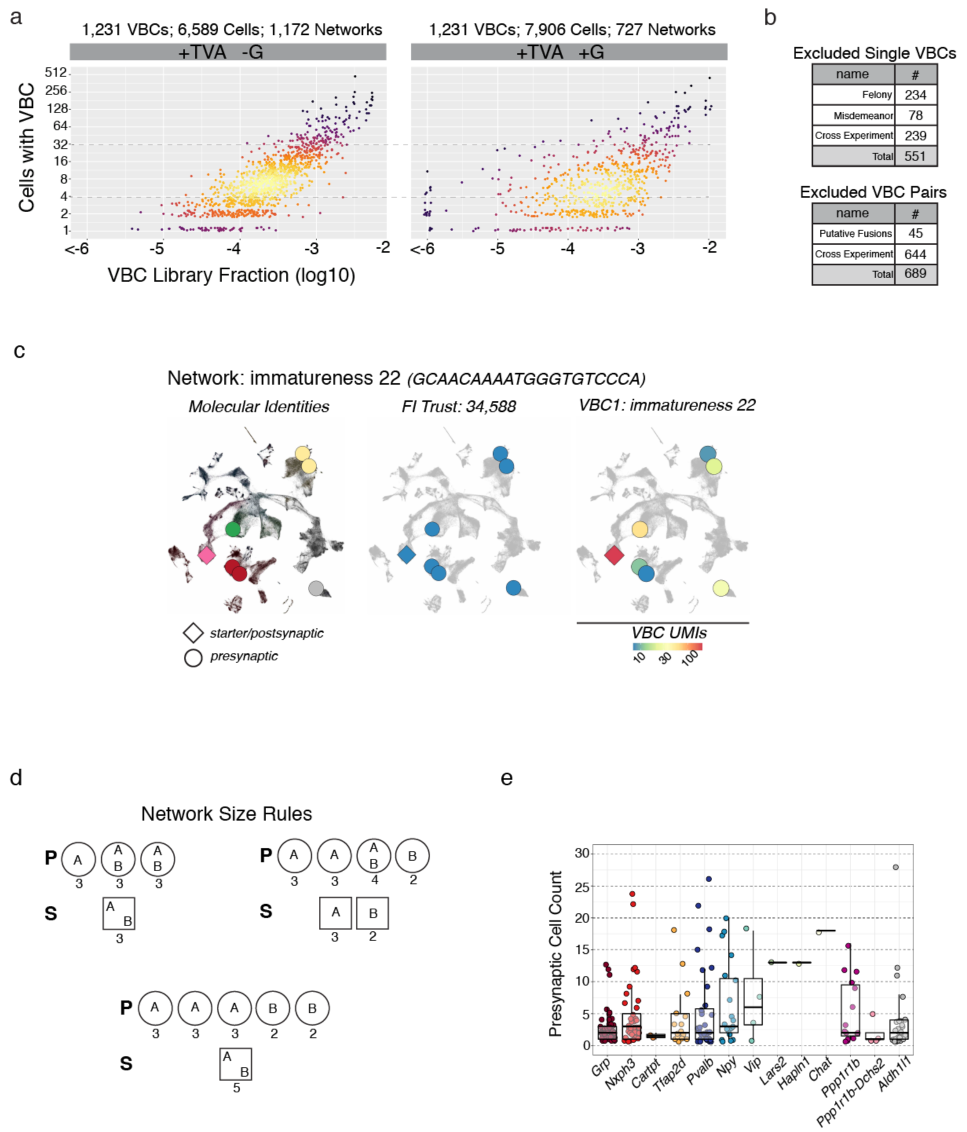

Excluded Single VBCs

name	#
Felony	234
Misdemeanor	78
Cross Experiment	239
Total	551

Excluded VBC Pairs

name	#
Putative Fusions	45
Cross Experiment	644
Total	689

Supplementary Figure 7. SBARRO inference of synaptic networks through VBC-based CIPs. a. The effect of rabies virus spread on the relationship between VBC library abundance and number of cells in which each VBC was ascertained. *Right*, a single experiment (“SCC07_1e3_A”) in which EnvA-RV*dG-EGFP*$_{VBC}$ founder infections were complemented with glycoprotein endowing monosynaptic retrograde spread (+G). *Left*, a version of the G- starter cell corpus (Fig. 2g), consisting exclusively of founder infections, randomly down-sampled to match equivalent VBC numbers (n=1,231). **b.** Tables of VBCs (*top*) and VBC pairs (*bottom*) excluded from network inference using EnvA-RV*dG-EGFP*$_{VBC}$ (Methods). **c.** Molecular identities, FI trust score and VBC UMI counts mapped onto the UMAP embedding for cells associated with the “immatureness 22” network. **d.** Schematic describing how the “Network Size” parameter was calculated for starter cells (squares) and presynaptic cells (circles) based on examples using two VBCS, “A” and “B”. Network Size values are listed below each cell. **e.** Inferred presynaptic network sizes by starter cell subtype for all identified starter cells. Network data include n=365 total networks identified across n=22 independent culture wells derived n=3 replicates of primary cell culture. Boxes define the interquartile range and whiskers delineate 1.5 times this range. Source data are provided as a Source Data file.

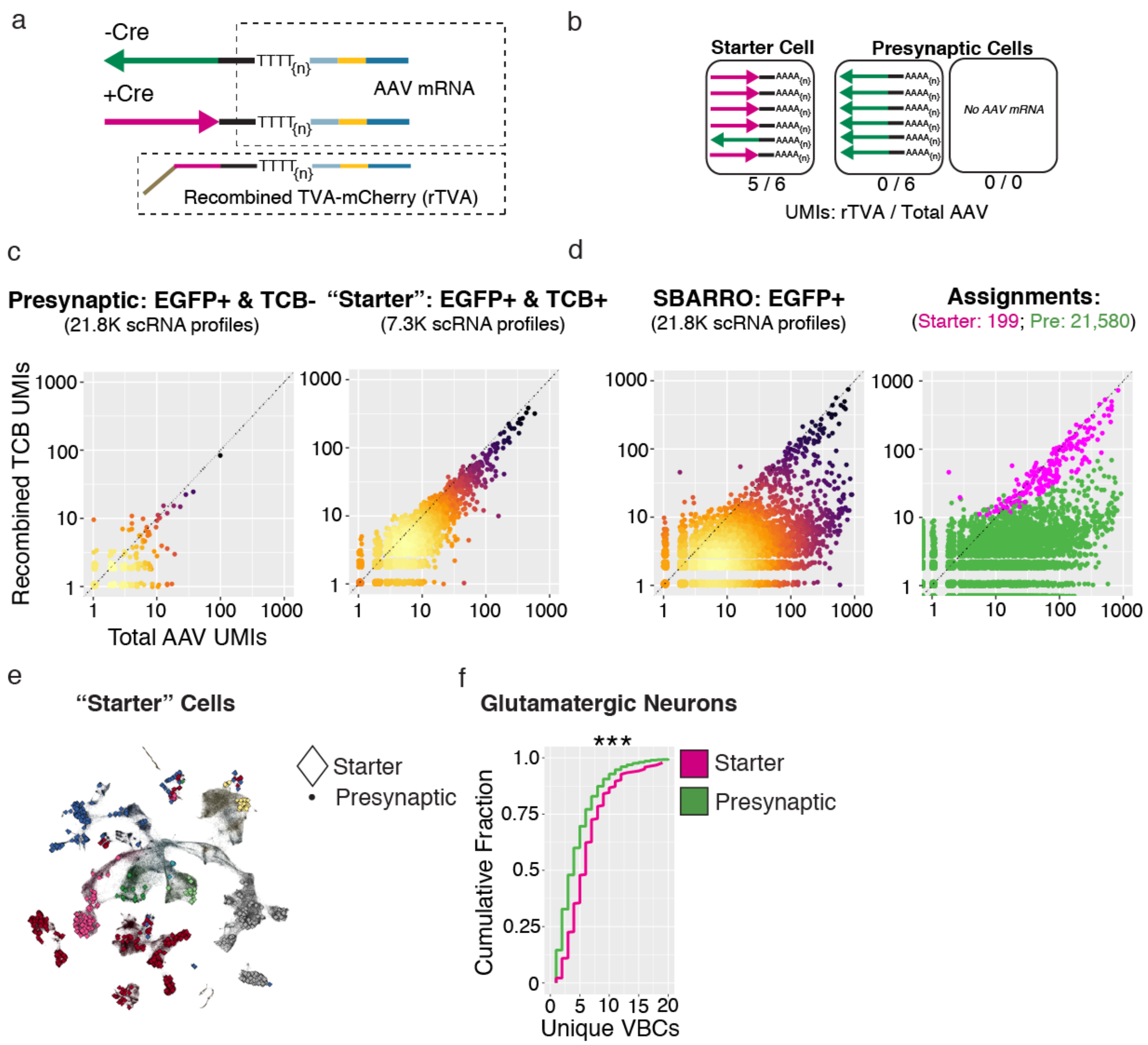

Supplementary Figure 8. Assigning starter cell identities to SBARRO scRNA profiles. a-c. Identifying starter cells. Starter cells get functional *TVA-mCherry* and *G* mRNAs after Cre-mediated recombination of CAG-Flex-TVA-mCherry and CAG-Flex-B19(G) rAAV genomes. **a**. 3' RNA schematic of recombined (+Cre, magenta) or unrecombined (-Cre, green) rAAV mRNAs after first strand synthesis. The sequenced region critical for determining gene identity – the black bar in the dashed upper box – is unaffected by recombination, thus the vast majority of single cell counts of rAAV mRNAs are not recombination-informative. To generate sequencing libraries selectively for recombined rAAV mRNAs, we amplified and independently sequenced recombined *TVA-mCherry* transcripts (bottom dashed box; Methods). **b**. Cartoon illustrating recombined versus unrecombined rAAV mRNA content of starter and presynaptic cells. Starter cell RNA profiles are enriched for recombined rAAV mRNAs, while presynaptic cells are enriched for unrecombined molecules or have no detectable rAAV mRNAs. **c**. Validation of rAAV mRNA signatures for starter and presynaptic cells after physical separation via FACS. Scatter plots comparing UMI counts of recombined *TVA-mCherry* mRNA vs Total rAAV mRNA for scRNA profiles resulting from FACS-based separation and independent library generation of presynaptic (*left*, EGFP+/TVA-mCherry-) or starter (*right*, EGFP+/TVA-mCherry+) cells. The dotted represents cell profiles for which all rAAV mRNAs are recombined *TVA-mCherry* transcripts. scRNA profiles from sorted starter cells have higher counts of Total rAAV mRNAs and those counts are largely from recombined *TVA-mCherry mRNAs*. Lighter colors indicate higher point densities. **d**. Assigning starter cell identities to SBARRO scRNA profiles (n=21,580) from a single experiment ("SCC07_1e2_C"). *Left*, scatterplot of recombined *TVA-mCherry* vs Total rAAV mRNA counts. *Right*, color-coded by starter (n=199) or presynaptic (n=21,580) assignment based on binomial testing for starter cells which exhibit statistical enrichments for recombined *TVA-mCherry* counts (versus Total rAAV

counts) and Total rAAV counts (versus all UMIs; Methods). **e**. UMAP locations of starter and presynaptic cells. **f**. Cumulative distribution of unique VBCs across glutamatergic neuron starter and presynaptic cells (*** = $p < 2.2e-16$, two-sided Kolmogorov–Smirnov Test). Data are from n=430 starter and n= 35,746 presynaptic cells derived from n=22 culture wells across n=3 primary culture replicates. Source data are provided as a Source Data file.

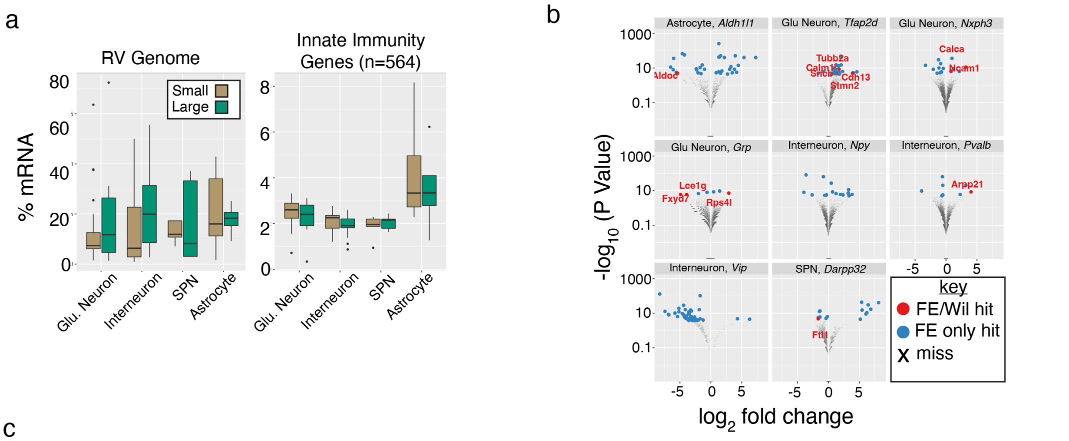

c

gene	cell_type	subtype_marker_gene	upregulated_in	n_cells_small_network_starter	n_cells_large_network_starter	log2_fold_change	fishers_exact_raw_p	n_genes_tested	fishers_exact_corrected_p	wilcoxon_p	n_hits_out_of_100_bootstrap_reps
Aldoc	Astrocyte	Aldh1l1	small_network_starter	9	4	5.397	1.20e−05	1179	1.41e−02	0.028	0
Calm1	Glu_Neuron	Tfap2d	large_network_starter	5	3	0.993	6.58e−10	1568	1.03e−06	0.036	1
Cdh13	Glu_Neuron	Tfap2d	large_network_starter	5	3	3.900	1.29e−05	1568	2.02e−02	0.026	1
Sncb	Glu_Neuron	Tfap2d	large_network_starter	5	3	1.323	1.68e−05	1568	2.63e−02	0.036	0
Stmn2	Glu_Neuron	Tfap2d	large_network_starter	5	3	1.246	1.11e−15	1568	1.74e−12	0.036	2
Tubb2a	Glu_Neuron	Tfap2d	large_network_starter	5	3	1.699	3.66e−09	1568	5.73e−06	0.036	1
Calca	Glu_Neuron	Nxph3	large_network_starter	17	7	3.233	5.52e−12	5069	2.80e−08	0.022	0
Ncam1	Glu_Neuron	Nxph3	large_network_starter	17	7	0.805	8.43e−07	5069	4.27e−03	0.013	3
Fxyd7	Glu_Neuron	Grp	small_network_starter	23	2	4.900	1.12e−06	4847	5.41e−03	0.050	0
Lce1g	Glu_Neuron	Grp	small_network_starter	23	2	3.919	1.72e−06	4847	8.32e−03	0.043	0
Rps4l	Glu_Neuron	Grp	large_network_starter	23	2	2.932	1.29e−07	4847	6.28e−04	0.014	0
Arpp21	Interneuron	Pvalb	large_network_starter	9	7	4.021	5.08e−09	3707	1.88e−05	0.044	1
Ftl1	SPN	Darpp32	small_network_starter	4	5	1.611	1.47e−05	1184	1.74e−02	0.016	2

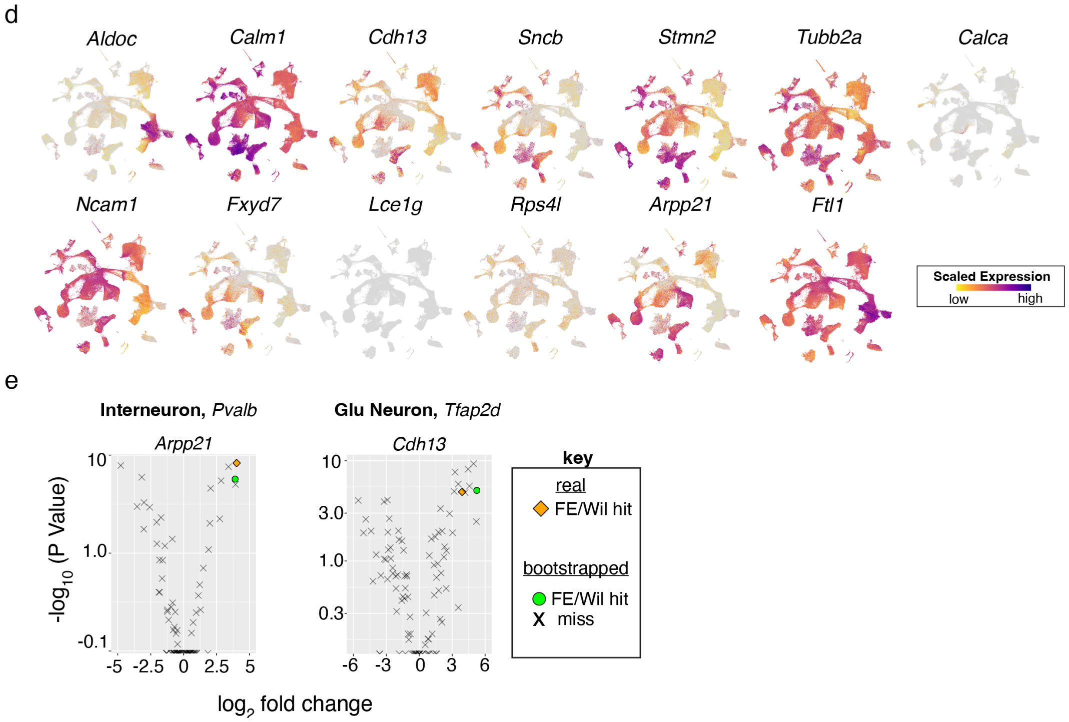

Supplementary Figure 9. Properties of postsynaptic starter cell infection and host cell RNAs associated with presynaptic network size inferences. a. Viral load (% of all mRNAs derived from 5 rabies virus genes; *left*) and innate immunity expression scores (aggregated from 584 curated genes[22]; *right*) across starter cell RNA profiles (n=144) did show detectable differences across four major brain cell types but not across "large" or "small" presynaptic network size groupings (Viral load: cell type, p = 0.10; presynaptic network size, p=0.05. Innate immune expression: cell type, p = 5.3e-12; presynaptic network size, p=0.16. Two-way ANOVA). **b.** Volcano plots illustrating results from differential expression testing of starter cell RNA profiles comparing "large" and "small" presynaptic network size categories by starter cell subtypes. UMI counts for each gene of sufficient expression were aggregated by inferred presynaptic network size category then compared (Fisher's Exact Test; Methods). Genes passing corrected p value thresholds ($p < 0.05$, blue dots) were further tested for differences in single-cell scaled expression (Wilcoxon Test; Methods). Those genes that pass this additional test ($p < 0.05$) were considered hits and labeled (red dots). **c**. Summary table describing differential expression results for those genes identified in b. **d**. Expression plots for the genes identified in

c. **e**. Volcano plots comparing differential expression results for *Arpp21* in *Pvalb*+ Interneurons (*left*) and *Cdh13* in *Tfap2d*+ Glutamatergic Neurons (*right*) in real data or 100 permuted replicates in which starter cell RNA profiles were randomly replaced by presynaptic profiles of the same subtype (Methods). The real data is shown with a gold diamond; permuted replicates passing aggregate UMI (Fisher's Exact Test) and scaled expression comparisons (two-sided Wilcoxon Test) are shown as green circles; all other comparisons are shown with grey crosses. Boxes define the interquartile range and whiskers delineate 1.5 times this range. Source data are provided as a Source Data file.

a

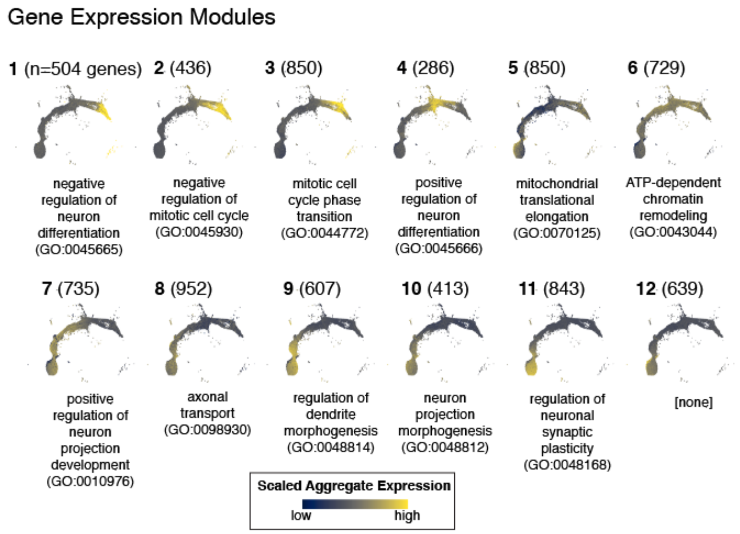

b

Gene Ontology (Biological Process)

"mitochondrial respiratory chain complex assembly" (GO:0033108) (n=41 of 97)

Coa3, Cox14, Cox18, Ecsit, Lyrm7, Ndufa1, Ndufa11, Ndufa3, Ndufa5, Ndufa6, Ndufa8, Ndufab1, Ndufaf3, Ndufaf5, Ndufaf7, Ndufb10, Ndufb2, Ndufb3, Ndufb4, Ndufb5, Ndufb8, Ndufb9, Ndufc1, Ndufs1, Ndufs3, Ndufs5, Ndufs7, Ndufv2, Ndufv3, Oxa1l, Sco2, Sdhaf2, Sdhaf4, Slc25a33, Surf1, Taz, Ttc19, Uqcc2, Uqcc3, Uqcr10, Uqcrb

"phagosome maturation" (GO:0090382) (n=15 of 27)

Atp6v0a1, Atp6v0b, Atp6v0d1, Atp6v0e2, Atp6v1a, Atp6v1b2, Atp6v1c1, Atp6v1d, Atp6v1e1, Atp6v1f, Atp6v1g2, Atp6v1h, Coro1a, Rab34, Rab43

"positive regulation of synaptic transmission" (GO:0050806) (n=24 of 340)

Arc, Cacng3, Clstn1, Clstn3, Dlg4, Dtnbp1, Hap1, Kctd13, Lgi1, Mpp2, Nlgn3, Nptn, Plk2, Prkcz, Pten, Ptk2b, Rgs14, Rims4, Shank3, Shisa7, Slc8a2, Sqstm1, Stx1b, Vamp2

SynGO

"synaptic vesicle cycle" (GO:0099504) (n=55 of 171)

Abi2, Ap1s2, Ap2b1, Ap2m1, Atp6ap1, Atp6v0a1, Atp6v0d1, Atp6v1b2, Atp6v1c1, Atp6v1d, Atp6v1e1, Atp6v1f, Atp6v1g2, Atp6v1h, Bin1, Cacnb4, Camk2a, Cltc, Cplx1, Dnajc5, Dnm1, Doc2b, Dtnbp1, Epn1, Htr1b, Napa, Napb, Nsf, Pacsin1, Prkar1b, Prkcb, Prrt2, Rab3a, Rab3b, Rapgef4, Rimbp2, Scamp5, Slc32a1, Snap25, Snca, Sncb, Stx1b, Stx3, Stxbp1, Sv2a, Sv2c, Syn1, Syn2, Syngr1, Syngr3, Synj1, Syp, Syt7, Vamp2, Vamp4

Supplementary Figure 10. Molecular correlates of rabies virus transmission during SPN development. a. Modules of gene expression (n=12) identified with Monocle3 (Methods). For each numbered module, the count of associated genes is shown parenthetically, module expression is color-coded by aggregate expression and a representative enrichment for biological process gene ontologies categories is shown (adjusted $p < 0.05$, one-sided Fisher's exact test with False Discovery Rate correction). **b.** Names of rabies virus-transmission correlated "synaptic vesicle cycle" genes (n=55 of 171 in SynGO category GO:0099504).
