# Supplementary material for: Ascertaining cells’ synaptic connections and RNA expression simultaneously with barcoded rabies virus libraries
Source: Nat Commun. 2022 Nov 16;13:6993. doi: 10.1038/s41467-022-34334-1 (PMC9668842; doi:10.1038/s41467-022-34334-1)
Supplement: Supplementary file 2 — Description of Additional Supplementary Files [file 41467_2022_34334_MOESM2_ESM.pdf]

Description of Additional Supplementary Files

File Name: Supplementary Data 1
Description: Oligonucleotide Guide

File Name: Supplementary Data 2
Description: Sequencing Summaries Across Experiments
