# Supplementary material for: Ascertaining cells’ synaptic connections and RNA expression simultaneously with barcoded rabies virus libraries
Source: Nat Commun. 2022 Nov 16;13:6993. doi: 10.1038/s41467-022-34334-1 (PMC9668842; doi:10.1038/s41467-022-34334-1)
Supplement: Supplementary file 5 — Reporting Summary [file 41467_2022_34334_MOESM5_ESM.pdf]

nature portfolio

Corresponding author(s): Steve McCarroll & Arpiar Saunders

Last updated by author(s): Oct 1, 2022

Reporting Summary

Nature Portfolio wishes to improve the reproducibility of the work that we publish. This form provides structure for consistency and transparency in reporting. For further information on Nature Portfolio policies, see our Editorial Policies and the Editorial Policy Checklist.

Statistics

For all statistical analyses, confirm that the following items are present in the figure legend, table legend, main text, or Methods section.

n/a	Confirmed	
☐	☒	The exact sample size (*n*) for each experimental group/condition, given as a discrete number and unit of measurement
☐	☒	A statement on whether measurements were taken from distinct samples or whether the same sample was measured repeatedly
☐	☒	The statistical test(s) used AND whether they are one- or two-sided<br>*Only common tests should be described solely by name; describe more complex techniques in the Methods section.*
☒	☐	A description of all covariates tested
☐	☒	A description of any assumptions or corrections, such as tests of normality and adjustment for multiple comparisons
☐	☒	A full description of the statistical parameters including central tendency (e.g. means) or other basic estimates (e.g. regression coefficient) AND variation (e.g. standard deviation) or associated estimates of uncertainty (e.g. confidence intervals)
☐	☒	For null hypothesis testing, the test statistic (e.g. *F*, *t*, *r*) with confidence intervals, effect sizes, degrees of freedom and *P* value noted<br>*Give P values as exact values whenever suitable.*
☒	☐	For Bayesian analysis, information on the choice of priors and Markov chain Monte Carlo settings
☒	☐	For hierarchical and complex designs, identification of the appropriate level for tests and full reporting of outcomes
☐	☒	Estimates of effect sizes (e.g. Cohen's *d*, Pearson's *r*), indicating how they were calculated

Our web collection on statistics for biologists contains articles on many of the points above.

Software and code

Policy information about availability of computer code

Data collection	No software was used for data collection.
Data analysis	RNA alignment and quantification was performed using a previously published workflow based on the STAR aligner (2.5.2a) and Drop-seq tools (v2.4.1)(Macosko et al. 2015). Three custom Java programs support the quantification of barcodes from viral libraries (plasmids & genomes) in addition to barcodes ascertained from mRNA of single cells: "TagReadWithRabiesBarcodes", "FilterValidRabiesBarcodes", "BipartiteRabiesVirusCollapse". All scripts are now publicly available as part of the Drop-seq tools release v2.4.1 which is available from Github (https://github.com/broadinstitute/Drop-seq/releases/tag/v2.4.1). Analysis of the scRNA-seq data was performed using lcaCluster (Saunders and Macosko et al. http://mccarrolllab.com/wp-content/uploads/2018/07/DropSeqlcaC1uster_2.0.tar), LIGER (0.4.2), Seurat (3.2.0), Monocle3(0.2.0), Panther(17.0), SynGO(release 20180731) and custom scripts written in the R programming language. Custom scripts are available upon request because they include more analyses then are included in the final publication.

For manuscripts utilizing custom algorithms or software that are central to the research but not yet described in published literature, software must be made available to editors and reviewers. We strongly encourage code deposition in a community repository (e.g. GitHub). See the Nature Portfolio guidelines for submitting code & software for further information.

Data

Policy information about availability of data

All manuscripts must include a data availability statement. This statement should provide the following information, where applicable:

- Accession codes, unique identifiers, or web links for publicly available datasets
- A description of any restrictions on data availability
- For clinical datasets or third party data, please ensure that the statement adheres to our policy

The raw and processed sequencing data reported in this paper are available from the GEO repository (Accession Code: GSE214386). The GRCm38.81 mouse genome build is publicly available (https://www.ncbi.nlm.nih.gov/assembly/GCF_000001635.20/). PantherDB(17.0; http://pantherdb.org/) and SynGO(release 20180731; https://www.syngoportal.org/) Source data are provided as a Source Data file for all main (Figures 1-5) and Supplemental Figures (Supplemental Data Figures 1-10).

Human research participants

Policy information about studies involving human research participants and Sex and Gender in Research.

Reporting on sex and gender	No human samples were used in this study
Population characteristics	No human samples were used in this study
Recruitment	No human samples were used in this study
Ethics oversight	No human samples were used in this study

Note that full information on the approval of the study protocol must also be provided in the manuscript.

Field-specific reporting

Please select the one below that is the best fit for your research. If you are not sure, read the appropriate sections before making your selection.

☒ Life sciences ☐ Behavioural & social sciences ☐ Ecological, evolutionary & environmental sciences

For a reference copy of the document with all sections, see nature.com/documents/nr-reporting-summary-flat.pdf

Life sciences study design

All studies must disclose on these points even when the disclosure is negative.

Sample size	Sample sizes for scRNA-seq datasets were not strictly predetermined through power analysis. Rather, previous analyses in our group suggested >50,000 scRNA-profiles were sufficient to describe molecular diversity from cortical mouse brain cells. To calibrate barcoded rabies libraries in single cells, we determined ~20,000 scRNA-profiles would enable careful characterization of viral libraries because abundant barcoded genomes would be sampled hundreds of times. To reconstruct hundreds to thousands of synaptic network - sample sizes which deemed appropriate for testing SBARRO technology - we generated uninfected control cell and rabies-infected datasets each of which contained ~145,000 scRNA profiles each. For all experiments, cells were sampled from at least three biological replicate of cultured primary cells generated at different times. For each time point, multiple replicates were ascertained from distinct culture wells.
Data exclusions	Individual RNA profiles were excluded for analysis if they were determined to be associated with multiple cells (cell-cell "doublets" as described in Saunders and Macosko et al. 2018). Excluded "doublets" represented only a small portion of the total cell libraries (<5%) and represent an accepted quality control step for such analyses.
Replication	The reproducibility was verified by ensuring differences reported from full datasets were replicated across > n=3 independent biological replicates.
Randomization	Experiments were controlled because individual cells from the same cell suspensions (generated from multiple embryonic mice / suspension) were randomly introduced into adjacent control culture wells or culture wells in which barcoded rabies was used to reconstruct synaptic networks.
Blinding	Blinding was not performed in these experiments because cells were sampled and compared from the same or adjacent culture in a systematic manner and subsequent analysis was all performed post-hoc.

Reporting for specific materials, systems and methods

We require information from authors about some types of materials, experimental systems and methods used in many studies. Here, indicate whether each material, system or method listed is relevant to your study. If you are not sure if a list item applies to your research, read the appropriate section before selecting a response.

Materials & experimental systems

n/a	Involved in the study	
☒	☐	Antibodies
☐	☒	Eukaryotic cell lines
☒	☐	Palaeontology and archaeology
☐	☒	Animals and other organisms
☒	☐	Clinical data
☒	☐	Dual use research of concern

Methods

n/a	Involved in the study	
☒	☐	ChIP-seq
☐	☒	Flow cytometry
☒	☐	MRI-based neuroimaging

Eukaryotic cell lines

Policy information about cell lines and Sex and Gender in Research

Cell line source(s)	HEK-293T/17 (ATCC, CRL-11268), BHK-EnvA (Columbia Univ. Zuckerman Virus Core)
Authentication	Cell lines were not authenticated
Mycoplasma contamination	All cell lines tested negative for mycoplasma
Commonly misidentified lines (See ICLAC register)	None

Animals and other research organisms

Policy information about studies involving animals; ARRIVE guidelines recommended for reporting animal research, and Sex and Gender in Research

Laboratory animals	Mouse. C57Blk6/N. Primary brain cell cultures derived from E16 male and female pups
Wild animals	No wild animals were used in this study
Reporting on sex	Findings apply to both sexes
Field-collected samples	No field-collected samples were used in this study
Ethics oversight	All procedures were approved by the Harvard Medical School Department of Comparative Medicine (DCM) and the Institutional Animal Care and Use Committee (IACUC)

Note that full information on the approval of the study protocol must also be provided in the manuscript.

Flow Cytometry

Plots

Confirm that:

☒ The axis labels state the marker and fluorochrome used (e.g. CD4-FITC).

☒ The axis scales are clearly visible. Include numbers along axes only for bottom left plot of group (a 'group' is an analysis of identical markers).

☒ All plots are contour plots with outliers or pseudocolor plots.

☒ A numerical value for number of cells or percentage (with statistics) is provided.

Methodology

Sample preparation	Primary brain cells expressing EGFP indicative of rabies virus infection were dissociated into a cell suspension.
Instrument	Mo Flo Astrios EQ cell sorter (Beckman Coulter; 70 um nozzle)
Software	FlowJo (version 10.8.1) was used for posthoc FACS analysis
Cell population abundance	~2-30% depending on the experiment. Purity was assessed by downstream scRNA-seq analysis.
Gating strategy	Cells suspensions were briefly sampled to determine the distribution of EGFP fluorescence. Gating was adjusted to acquire

Gating strategy

EGFP cells. Importantly, FACS enrichment was necessary but not sufficient to include cells in a viral barcode based network analysis because all sorted cells were analyzed with scRNA-seq. Thus gating was liberal.

☒ Tick this box to confirm that a figure exemplifying the gating strategy is provided in the Supplementary Information.
